# Supplementary material for: Molecular Markers in Maternal Blood Exosomes Allow Early Detection of Fetal Alcohol Spectrum Disorders
Source: Int J Mol Sci. 2022 Dec 21;24(1):135. doi: 10.3390/ijms24010135 (PMC9820501; doi:10.3390/ijms24010135)
Supplement: Supplementary file 1 [file ijms-24-00135-s001.zip › ijms-2006613-supplementary.pdf]

Table S1. Diagnostic criteria for the four syndromes comprising fetal alcohol spectrum disorders (FASD; adapted from Hoyme et al, 2016).

FASD			
**Fetal Alcohol Syndrome (FAS)**	**Partial Fetal Alcohol Syndrome (PFAS)**	**Alcohol-Related Neurodevelopmental Disorder (ARND)**	**Alcohol-Related Birth Defects (ARBD)**, which requires features **A** and **B**:
**A. Characteristic pattern of minor facial anomalies**, including at least 2 of the following:	**A.** A characteristic pattern of minor facial anomalies, including ≥2 of the following:	**A.** Documented prenatal alcohol exposure	**A.** Documented prenatal alcohol exposure
**B. Prenatal and/or postnatal growth deficiency.** Height and/or weight ≤10th percentile (plotted on a racially or ethnically appropriate growth curve).	**B.** Neurobehavioral impairments<br>c. Recurrent nonfebrile seizures (other causes of seizures having been ruled out).	**B.** Neurobehavioral impairments	**B.** One or more specific major malformations demonstrated in animal models and human studies to be the result of prenatal alcohol exposure.
**C. Deficient brain growth, abnormal morphogenesis, or abnormal neurophysiology**, including at least 1 of the following (below)	**C.** Neurobehavioral impairments		
**D. Neurobehavioral impairments.**			

i) Fetal Alcohol Syndrome (FAS). For children with documented prenatal alcohol exposure, the diagnosis requires all of the following features (**A–D**):

A. Characteristic pattern of minor facial anomalies, including at least 2 of the following:

1. Short palpebral fissures (≤10th centile)
2. Thin vermilion border of the upper lip (rank 4 or 5 on a racially normed lip/philtrum guide)
3. Smooth philtrum (rank 4 or 5 on a racially normed lip/philtrum guide, if available).

B. Prenatal and/or postnatal growth deficiency. Height and/or weight ≤10th percentile (plotted on a racially or ethnically appropriate growth curve).

C. Deficient brain growth, abnormal morphogenesis, or abnormal neurophysiology, including at least 1 of the following:

1. Head circumference equal to or less than the10th percentile

2. Structural brain anomalies

3. Recurrent nonfebrile seizures (other causes of seizures having been ruled out).

D. Neurobehavioral impairments.

1. For children at least 3 years old (a or b):

a. With Cognitive Impairment; evidence of global impairment (general conceptual ability at least 1.5 SD below the mean; or performance IQ or verbal IQ or spatial IQ at least 1.5 SD below the mean); or cognitive deficit in at least 1 neurobehavioral domain at least 1.5 SD below the mean (executive functioning, specific learning impairment, memory impairment or visual-spatial impairment).

b. With Behavioral Impairment But Without Cognitive Impairment; evidence of behavioral deficit in at least 1 domain at least 1.5 SD below the mean in impairments of self-regulation (mood or behavioral regulation impairment, attention deficit, or poor impulse control).

2. For children less than 3 years old: Evidence of developmental delay ≥1.5 SD below the mean.

ii) Partial Fetal Alcohol Syndrome (PFAS), for children with documented prenatal alcohol exposure, a diagnosis of PFAS requires features **A** and **B**:

A. A characteristic pattern of minor facial anomalies, including ≥2 of the following:

1. Short palpebral fissures (≤10th centile)

2. Thin vermilion border of the upper lip (rank 4 or 5 on a racially normed lip/philtrum guide)

3. Smooth philtrum (rank 4 or 5 on a racially normed lip/philtrum guide, if available).

B. Neurobehavioral impairments

1. For children at least 3 years old (a or b):

a. With Cognitive Impairment: Evidence of global impairment (general conceptual ability at least 1.5 SD below the mean, or performance IQ or verbal IQ or spatial IQ at least 1.5 SD below the mean); or cognitive deficit in at least 1 neurobehavioral domain at least 1.5 SD below the mean (executive functioning, specific learning impairment, memory impairment or

visual-spatial impairment).

b. With Behavioral Impairment Without Cognitive Impairment: Evidence of behavioral deficit in one or more domain, at least 1.5 SD below the mean in impairments of self-regulation (mood or behavioral regulation impairment, attention deficit, or impulse control).

2. For children less than 3 years old: Evidence of developmental delay at least 1.5 SD below the mean. For children without documented prenatal alcohol exposure, a diagnosis of PFAS requires all features, **A–C**:

A. A characteristic pattern of minor facial anomalies, including at least 2 of the following:

1. Short palpebral fissures (≤10th centile)

2. Thin vermilion border of the upper lip (rank 4 or 5 on a racially normed lip/philtrum guide, if available)

3. Smooth philtrum (rank 4 or 5 on a racially normed lip/philtrum guide, if available).

B. Growth deficiency or deficient brain growth, abnormal morphogenesis, or abnormal neurophysiology

1. Height and/or weight at or below the 10th percentile (plotted on a racially or ethnically appropriate growth curve, if available)

or

2. Deficient brain growth, abnormal morphogenesis or neurophysiology, including at least 1 of the following:

a. Head circumference at or below the 10th percentile

b. Structural brain anomalies

c. Recurrent nonfebrile seizures (other causes of seizures having been ruled out).

C. Neurobehavioral impairments

1. For children at least 3 years old (a or b):

a. With Cognitive Impairment: Evidence of global impairment (general conceptual ability at least 1.5 SD below the mean, or performance IQ or verbal IQ or spatial IQ at least 1.5 SD below the mean); or cognitive deficit in 1 or more neurobehavioral domain at least 1.5 SD below the mean (executive functioning, specific learning impairment, memory impairment, or visual-spatial impairment).

b. With Behavioral Impairment But Without Cognitive Impairment: Evidence of behavioral deficit in 1 or more domain at least 1.5 SD below the mean in impairments of self-regulation (mood or behavioral regulation impairment, attention deficit, or impaired impulse control).

2. For children less than 3 years old: Evidence of developmental delay at least 1.5 SD below the mean.

iii) Alcohol-Related Neurodevelopmental Disorder (ARND), which requires features **A** and **B** (this diagnosis cannot be made definitively in children <3 y of age):

A. Documented prenatal alcohol exposure

B. Neurobehavioral impairments

1. For children at least 3 years old (a or b):

a. With Cognitive Impairment: Evidence of global impairment (general conceptual ability at least 1.5 SD below the mean, or performance IQ or verbal IQ or spatial IQ ≥1.5 SD); or cognitive deficit in 2 or more neurobehavioral domains at least 1.5 SD below the mean (executive functioning, specific learning impairment, memory impairment or visual-spatial impairment)

b. With Behavioral Impairment Without Cognitive Impairment: Evidence of behavioral deficit in 2 or more domains at least 1.5 SD below the mean in impairments of self-regulation (mood or behavioral regulation impairment, attention deficit, or impulse control).

iv) Alcohol-Related Birth Defects (ARBD), which requires features **A** and **B**:

A. Documented prenatal alcohol exposure

B. One or more specific major malformations demonstrated in animal models and human studies to be the result of prenatal alcohol exposure. *Cardiac:* atrial septal defects, aberrant great vessels, ventricular septal defects, heart defects. *Skeletal:* radioulnar synostosis, vertebral segmentation defects, large joint contractures, scoliosis. *Renal:* aplastic/hypoplastic/dysplastic kidneys, "horseshoe" kidneys/ureteral duplications. *Eye:* strabismus, ptosis, retinal vascular anomalies, optic nerve hypoplasia. *Ear:* conductive hearing loss, neurosensory hearing loss.
